# Tratamento da Insuficiência Cardíaca no Brasil: Quando Estudo de Custo-Efetividade e Dados de Acesso Público às Terapias se Encontram

**DOI:** 10.36660/abc.20260314

**Published:** 2026-08-31

**Authors:** Henrique Iahnke Garbin, Fernando Luis Scolari, Andreia Biolo

**Affiliations:** 1 Universidade Federal do Rio Grande do Sul Faculdade de Medicina Porto Alegre RS Brasil Universidade Federal do Rio Grande do Sul – Faculdade de Medicina, Porto Alegre, RS – Brasil; 2 Hospital de Clinicas de Porto Alegre Porto Alegre RS Brasil Hospital de Clinicas de Porto Alegre, Porto Alegre, RS – Brasil

**Keywords:** Insuficiência Cardíaca, Análise de Custo-Efetividade, Brasil

A insuficiência cardíaca (IC) permanece sendo uma das principais causas de hospitalização e mortalidade cardiovascular no Brasil. Nos últimos anos, novas terapias farmacológicas modificadoras de prognóstico têm demonstrado reduzir mortalidade cardiovascular, hospitalizações por IC e melhorar a qualidade de vida. Nesse contexto, a disponibilidade dessas terapias no sistema público de saúde se torna um fator determinante para melhorar os desfechos clínicos em nível populacional. A recente revisão sistemática sobre custo-efetividade das terapias para IC no Brasil demonstrou que diversas intervenções farmacológicas, incluindo antagonistas do receptor mineralocorticoide, inibidores do cotransportador sódio-glicose 2 (ISGLT2) e inibidores do receptor de angiotensina-neprilisina, apresentam elevada atratividade econômica no cenário nacional. A dapagliflozina, por exemplo, apresentou razão de custo-efetividade incremental de Int$ 9.000/QALY, sendo considerada custo-efetiva.^1^

De forma interessante, evidências populacionais brasileiras reforçam o impacto clínico dessas estratégias, indo ao encontro do estudo de custo-efetividade. Uma análise avaliou a evolução temporal de mortalidade cardiovascular e hospitalizações por IC após a implementação do programa de acesso público à dapagliflozina no Brasil.^2^ A política foi inicialmente aprovada para pacientes com diabetes mellitus tipo 2 ou doença cardiovascular estabelecida com idade ≥ 65 anos. A medicação passou a ser disponibilizada também para pacientes ≥ 18 anos com IC com fração de ejeção reduzida, diabetes mellitus tipo 2 ou doença renal crônica, mediante laudo de medicamento especializado. O estudo populacional demonstrou redução nas tendências de mortalidade cardiovascular (redução de 6,6% em 2022, 4,7% em 2023 e 5,9% em 2024 comparado com 2017) e de hospitalizações por IC (redução de 23,8% em 2024 comparado com 2017) após a disponibilização e acesso à medicação em pacientes com idade ≥ 65 anos.^2^

Apesar desses avanços, o Brasil ainda carece de evidências nacionais de custo-efetividade para terapias cardiovasculares. Novos tratamentos para IC e cardiomiopatias, com benefício comprovado, permanecem com acesso restrito no sistema público de saúde.^3,4^ Assim, estudos como a revisão sistemática apresentada são fundamentais para orientar políticas de incorporação tecnológica e ampliar o acesso a terapias eficazes. A monitorização do acesso, integrada a dados populacionais e estratégias de saúde pública, é fundamental para reduzir lacunas terapêuticas, com impacto na melhora da qualidade de vida e na redução de hospitalizações e da mortalidade cardiovascular.
