# Supplementary material for: Tratamento da Insuficiência Cardíaca no Brasil: Quando Estudo de Custo-Efetividade e Dados de Acesso Público às Terapias se Encontram
Source: Arq Bras Cardiol. 2026 Aug 31;123(8):e20260314. [Article in Portuguese] doi: 10.36660/abc.20260314 (PMC13671142; doi:10.36660/abc.20260314)
Supplement: Translation

# Translation: Treatment of Heart Failure in Brazil: Where Cost-Effectiveness Studies and Public Access Data to Therapies Meet

**DOI:** 10.36660/abc.20260314i

**Authors:** Henrique Iahnke Garbin, Fernando Luis Scolari, Andreia Biolo

**Affiliations:** 1 Porto Alegre RS Brazil Universidade Federal do Rio Grande do Sul – Faculdade de Medicina, Porto Alegre, RS – Brazil; 2 Porto Alegre RS Brazil Hospital de Clinicas de Porto Alegre, Porto Alegre, RS – Brazil

**Keywords:** Heart Failure, Cost-Effectiveness Analysis, Brazil

Heart failure (HF) remains a leading cause of hospitalization and cardiovascular mortality in Brazil. In recent years, new prognostically modifying pharmacological therapies have been shown to reduce cardiovascular mortality, HF hospitalizations, and improve quality of life. In this context, the availability of these therapies in the public health system becomes a determining factor in improving clinical outcomes at the population level. A recent systematic review on the cost-effectiveness of HF therapies in Brazil demonstrated that several pharmacological interventions, including mineralocorticoid receptor antagonists, sodium-glucose cotransporter 2 (SGLT2) inhibitors, and angiotensin-neprilysin receptor inhibitors, have high economic attractiveness in the national scenario. Dapagliflozin, for example, showed an incremental cost-effectiveness ratio of Int$ 9,000/QALY, and is considered cost-effective.^1^

Interestingly, Brazilian population-based evidence reinforces the clinical impact of these strategies, supporting the cost-effectiveness study. One analysis evaluated the temporal evolution of cardiovascular mortality and hospitalizations for heart failure after the implementation of the public access program for dapagliflozin in Brazil.^2^ The policy was initially approved for patients with type 2 diabetes mellitus or established cardiovascular disease aged ≥ 65 years. The medication was also made available to patients ≥ 18 years with heart failure with reduced ejection fraction, type 2 diabetes mellitus, or chronic kidney disease, upon presentation of a specialized medication report. The population-based study demonstrated a reduction in cardiovascular mortality trends (a 6.6% reduction in 2022, 4.7% in 2023, and 5.9% in 2024 compared to 2017) and hospitalizations for heart failure (a 23.8% reduction in 2024 compared to 2017) after the availability and access to medication in patients aged ≥ 65 years.^2^

Despite these advances, Brazil still lacks national evidence of cost-effectiveness for cardiovascular therapies. New treatments for heart failure and cardiomyopathies, with proven benefits, remain with restricted access in the public health system.^3,4^ Thus, studies such as the systematic review presented are fundamental to guiding policies for technological incorporation and expanding access to effective therapies. Monitoring access, integrated with population data and public health strategies, is essential to reduce therapeutic gaps, impacting the improvement of quality of life and the reduction of hospitalizations and cardiovascular mortality.
