# Supplementary material for: Tratamento da Insuficiência Cardíaca no Brasil: Quando Estudo de Custo-Efetividade e Dados de Acesso Público às Terapias se Encontram
Source: Arq Bras Cardiol. 2026 Aug 31;123(8):e20260314. [Article in Portuguese] doi: 10.36660/abc.20260314 (PMC13671142; doi:10.36660/abc.20260314)
Supplement: Related texts

# Reply: Carta-resposta

**Authors:** Eduardo Gehling Bertoldi, Guilherme Augusto Reissig Pereira, Mariana de Castro Lopes, Bernardo Ludwig Dama, Carisi Anne Polanczyk

**Affiliations:** 1 Hospital de Clínicas de Porto Alegre Porto Alegre RS Brasil Hospital de Clínicas de Porto Alegre, Porto Alegre, RS – Brasil; 2 Universidade Federal de Pelotas Pelotas RS Brasil Universidade Federal de Pelotas, Pelotas, RS – Brasil; 3 Universidade Federal do Rio Grande do Sul Porto Alegre RS Brasil Universidade Federal do Rio Grande do Sul, Porto Alegre, RS – Brasil

Agradecemos aos autores da carta pelo interesse em nossa revisão sistemática e pela oportunidade de ampliar a discussão sobre o tratamento da insuficiência cardíaca no Brasil. Nosso artigo^1^ teve como objetivo específico sintetizar as evidências nacionais de custo-efetividade de terapias farmacológicas e não-farmacológicas para insuficiência cardíaca, sob a perspectiva do SUS. Nesse sentido, embora o assunto principal da carta, que é o acesso efetivo às terapias após sua incorporação, não tenha sido diretamente investigado em nossa revisão, concordamos que é um tema complementar e essencial para que o valor estimado em modelos econômicos se traduza em benefício clínico populacional.

A incorporação de uma tecnologia custo-efetiva representa apenas uma etapa do ciclo de avaliação em saúde. A geração de valor depende também de disponibilidade, critérios de elegibilidade, prescrição adequada, e capacidade de monitorar desfechos em vida real. Os dados populacionais citados na carta, relacionados ao acesso público à dapagliflozina, ilustram a importância de acompanhar tendências de hospitalizações e mortalidade após políticas de incorporação. Ao mesmo tempo, tais análises devem ser interpretadas com cautela, por sua natureza observacional e pela possibilidade de influência de fatores temporais e assistenciais.

Portanto, concordamos que avaliações econômicas nacionais devem ser progressivamente integradas a estudos de implementação, análises de impacto orçamentário, dados de utilização e monitoramento pós-incorporação. Essa agenda é particularmente relevante em insuficiência cardíaca, condição de alta carga clínica e econômica, na qual intervenções eficazes e custo-efetivas podem ter impacto substancial, desde que o acesso seja equitativo e sustentável. Assim, entendemos que a carta reforça uma mensagem central de nossa revisão: ampliar a produção e o uso de evidências nacionais é fundamental para orientar decisões de incorporação, qualificar políticas públicas e melhorar os desfechos dos pacientes com insuficiência cardíaca no Brasil.

## References

[1] 1 Pereira GAR, Lopes MC, Dama BL, Polanczyk CA, Bertoldi EG. Cost-Effectiveness of Therapies for Heart Failure in Brazil: A Systematic Review. Arq Bras Cardiol. 2026;123(3):e20250712. doi: 10.36660/abc.20250712.10.36660/abc.20250712PMC1312821142018787

# Reply

**Authors:** Eduardo Gehling Bertoldi, Guilherme Augusto Reissig Pereira, Mariana de Castro Lopes, Bernardo Ludwig Dama, Carisi Anne Polanczyk

**Affiliations:** 1 Porto Alegre RS Brazil Hospital de Clínicas de Porto Alegre, Porto Alegre, RS – Brazil; 2 Pelotas RS Brazil Universidade Federal de Pelotas, Pelotas, RS – Brazil; 3 Porto Alegre RS Brazil Universidade Federal do Rio Grande do Sul, Porto Alegre, RS – Brazil

We thank the authors of the letter for their interest in our systematic review and for the opportunity to broaden the discussion on the treatment of heart failure in Brazil. Our article^1^ had the specific objective of synthesizing national evidence on the cost-effectiveness of pharmacological and non-pharmacological therapies for heart failure, from the perspective of the Brazilian Unified Health System (SUS). In this sense, although the main subject of the letter, which is effective access to therapies after their incorporation, was not directly investigated in our review, we agree that it is a complementary and essential theme for the value estimated in economic models to translate into clinical benefit for the population.

The incorporation of cost-effective technology represents only one step in the health evaluation cycle. Value generation also depends on availability, eligibility criteria, appropriate prescription, and the ability to monitor outcomes in real-world scenarios. The population data cited in the letter, related to public access to dapagliflozin, illustrate the importance of monitoring trends in hospitalizations and mortality after incorporation policies. At the same time, such analyses should be interpreted with caution, due to their observational nature and the possibility of influence from temporal and care-related factors.

Therefore, we agree that national economic evaluations should be progressively integrated with implementation studies, budget impact analyses, utilization data, and post-incorporation monitoring. This agenda is particularly relevant in heart failure, a condition with a high clinical and economic burden, where effective and cost-effective interventions can have a substantial impact, provided that access is equitable and sustainable. Thus, we understand that the letter reinforces a central message of our review: expanding the production and use of national evidence is fundamental to guiding incorporation decisions, improving public policies, and enhancing outcomes for patients with heart failure in Brazil.
